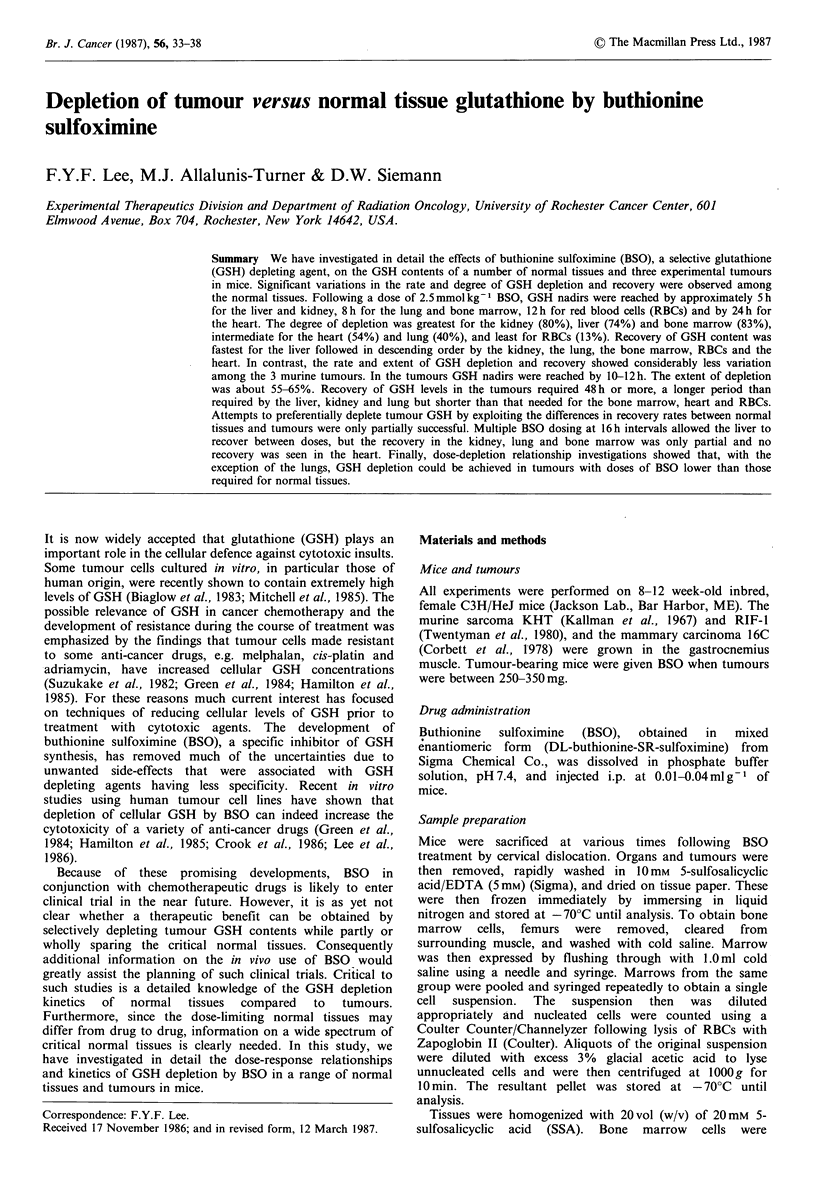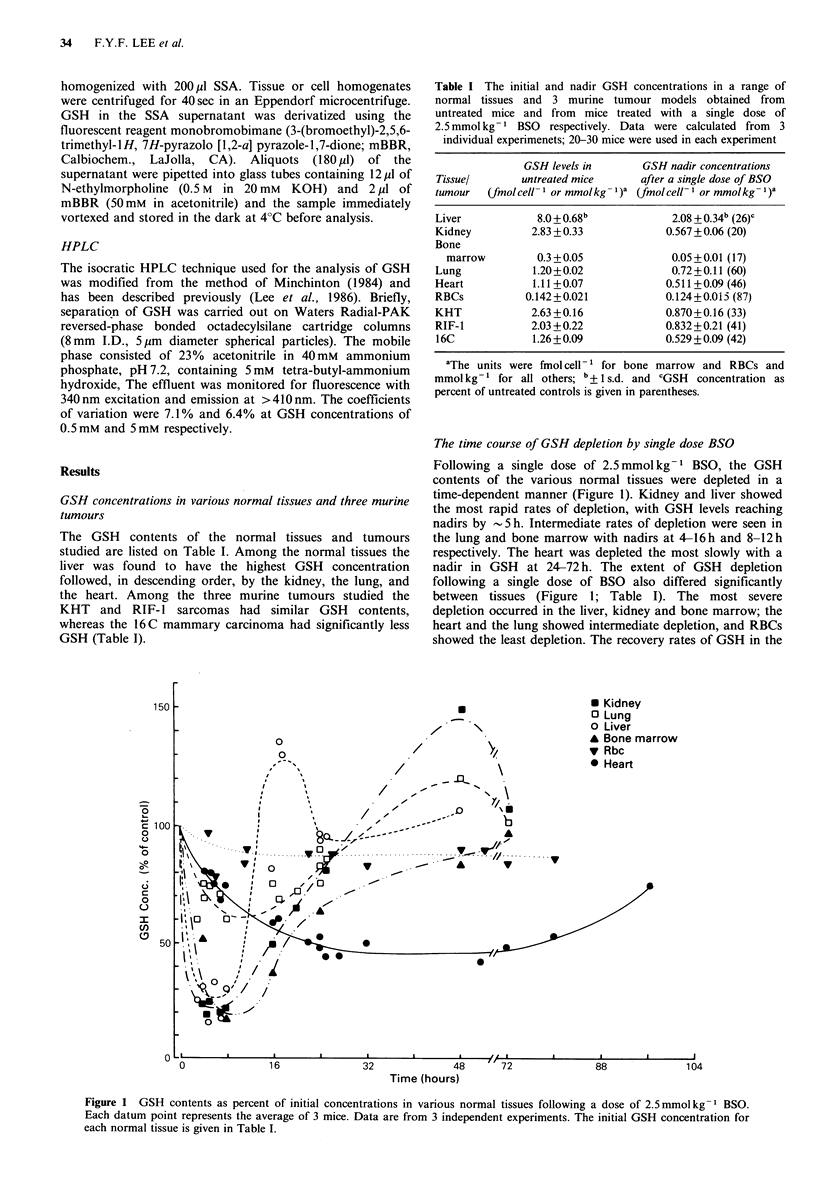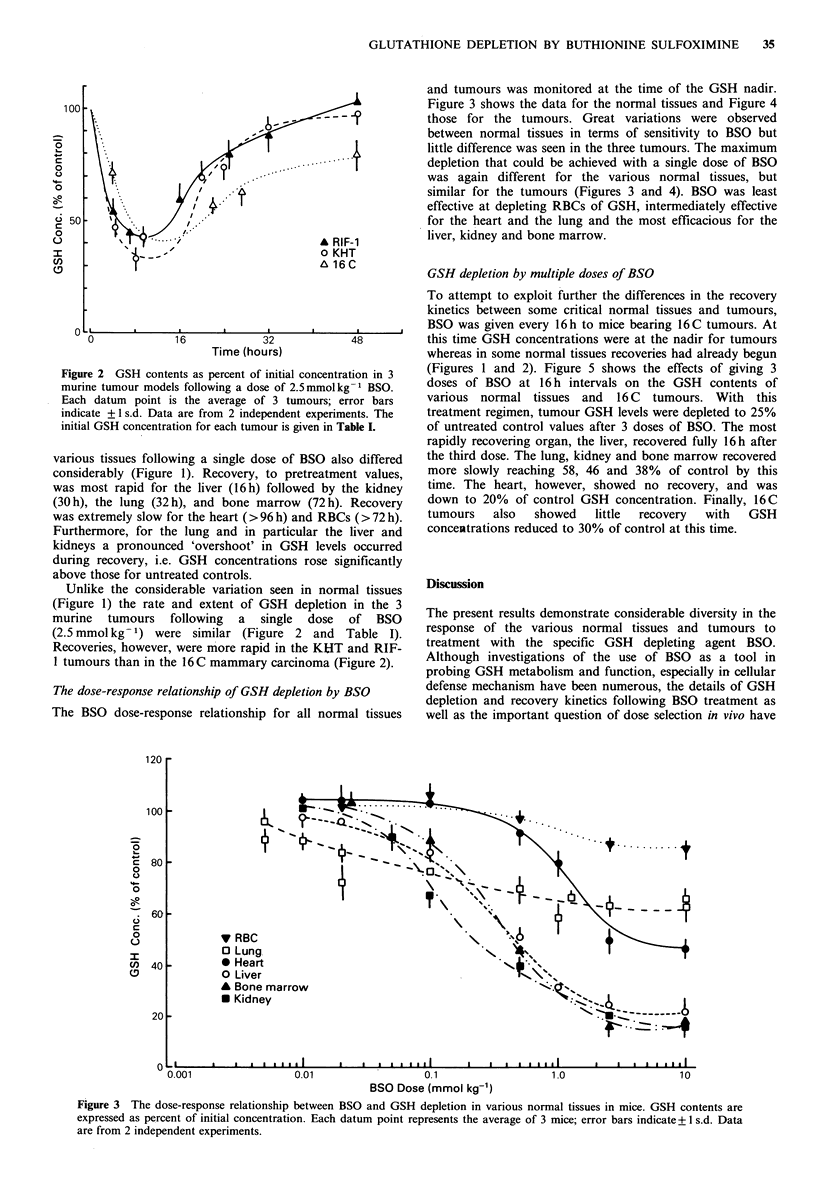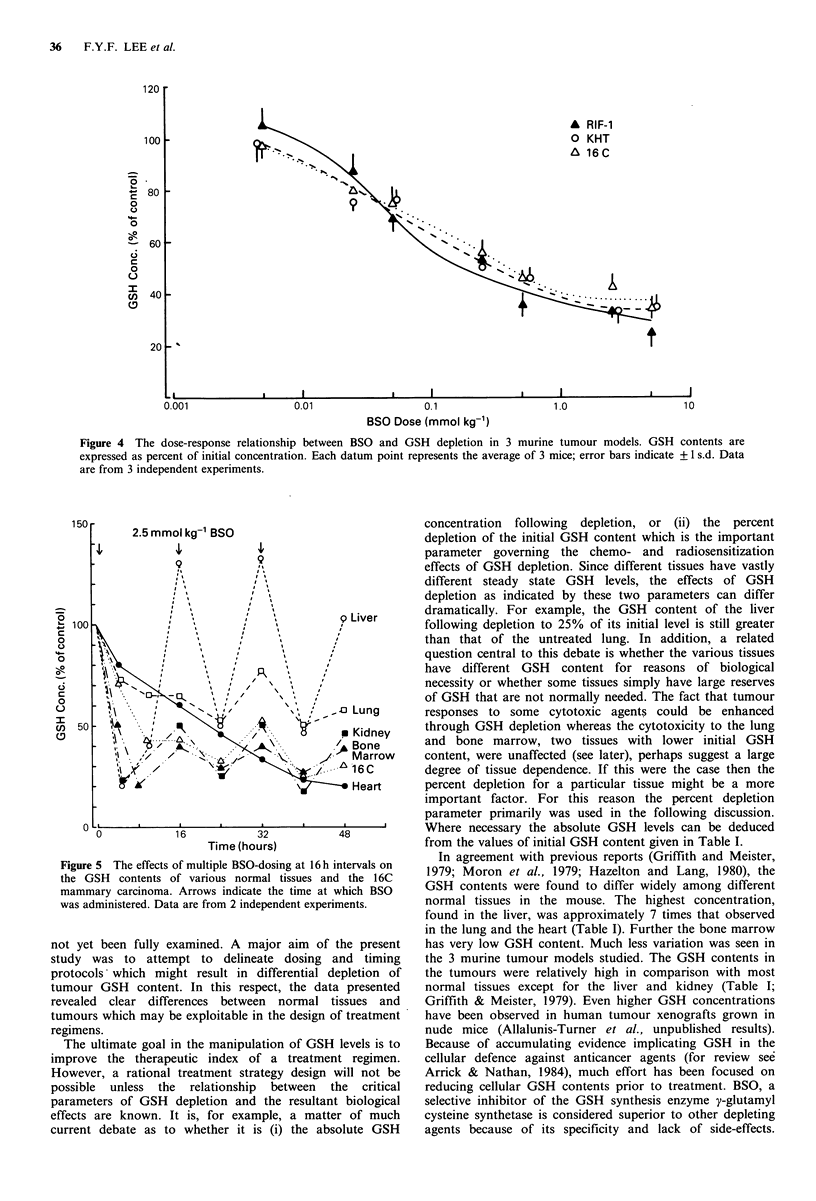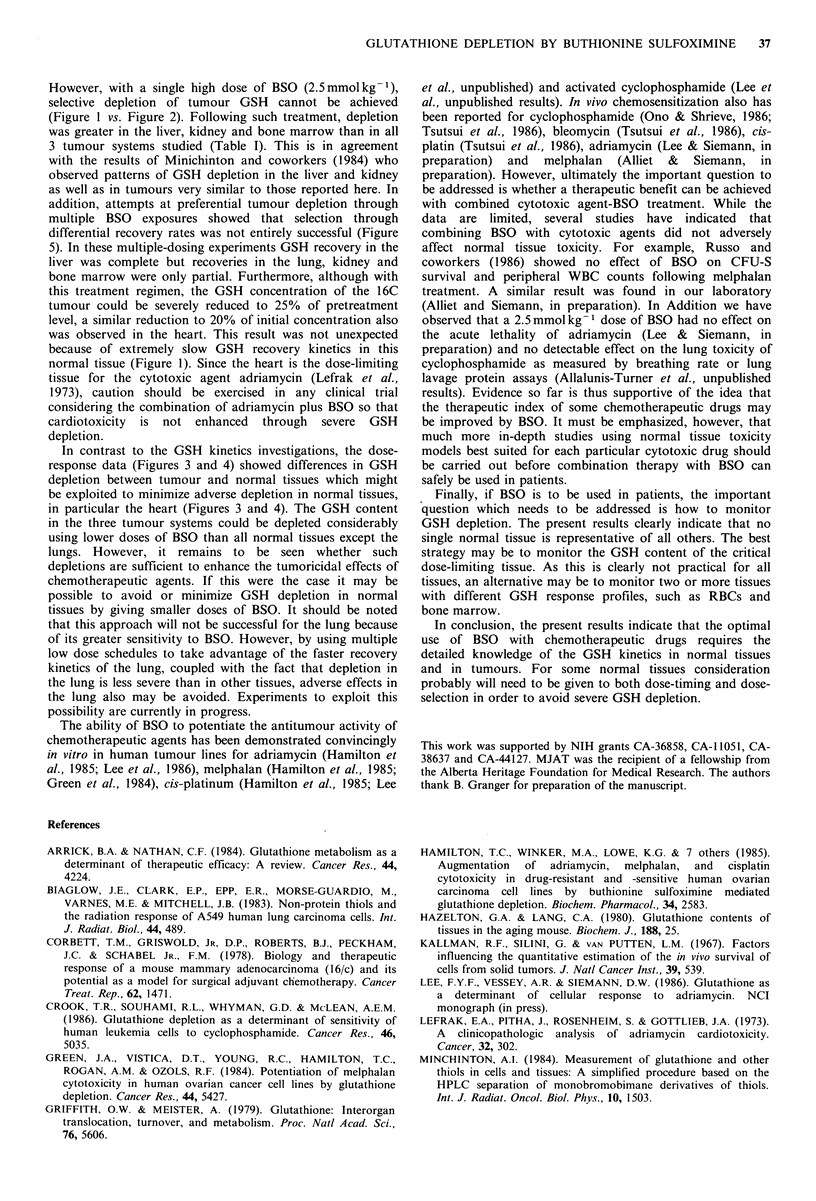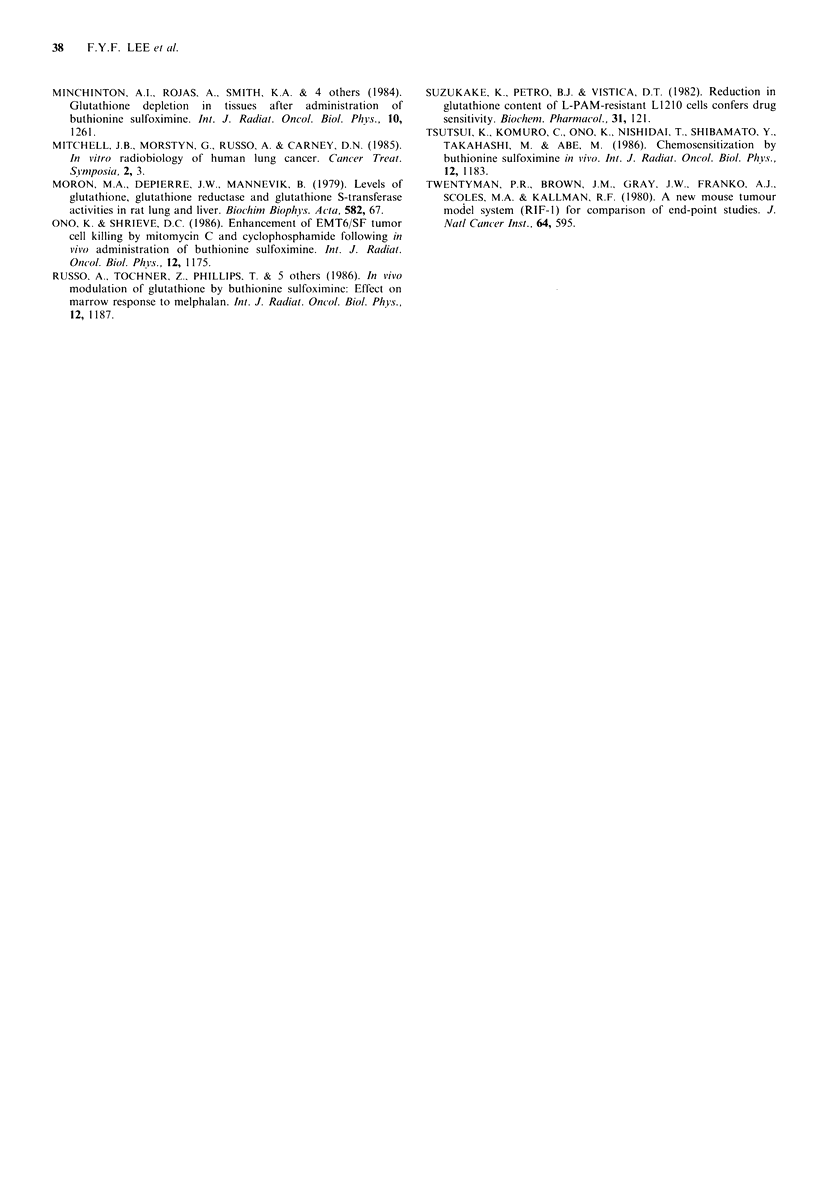# Depletion of tumour versus normal tissue glutathione by buthionine sulfoximine.

**DOI:** 10.1038/bjc.1987.148

**Published:** 1987-07

**Authors:** F. Y. Lee, M. J. Allalunis-Turner, D. W. Siemann

## Abstract

We have investigated in detail the effects of buthionine sulfoximine (BSO), a selective glutathione (GSH) depleting agent, on the GSH contents of a number of normal tissues and three experimental tumours in mice. Significant variations in the rate and degree of GSH depletion and recovery were observed among the normal tissues. Following a dose of 2.5 mmol kg-1 BSO, GSH nadirs were reached by approximately 5 h for the liver and kidney, 8 h for the lung and bone marrow, 12 h for red blood cells (RBCs) and by 24 h for the heart. The degree of depletion was greatest for the kidney (80%), liver (74%) and bone marrow (83%), intermediate for the heart (54%) and lung (40%), and least for RBCs (13%). Recovery of GSH content was fastest for the liver followed in descending order by the kidney, the lung, the bone marrow, RBCs and the heart. In contrast, the rate and extent of GSH depletion and recovery showed considerably less variation among the 3 murine tumours. In the tumours GSH nadirs were reached by 10-12 h. The extent of depletion was about 55-65%. Recovery of GSH levels in the tumours required 48 h or more, a longer period than required by the liver, kidney and lung but shorter than that needed for the bone marrow, heart and RBCs. Attempts to preferentially deplete tumour GSH by exploiting the differences in recovery rates between normal tissues and tumours were only partially successful. Multiple BSO dosing at 16 h intervals allowed the liver to recover between doses, but the recovery in the kidney, lung and bone marrow was only partial and no recovery was seen in the heart. Finally, dose-depletion relationship investigations showed that, with the exception of the lungs, GSH depletion could be achieved in tumours with doses of BSO lower than those required for normal tissues.


© The Macmillan Press Ltd., 1987

### Depletion of tumour *versus* normal tissue glutathione by buthionine sulfoximine

F.Y.F. Lee, M.J. Allalunis-Turner & D.W. Siemann

*Experimental Therapeutics Division and Department of Radiation Oncology, University of Rochester Cancer Center, 601 Elmwood Avenue, Box 704, Rochester, New York 14642, USA.*

**Summary** We have investigated in detail the effects of buthionine sulfoximine (BSO), a selective glutathione (GSH) depleting agent, on the GSH contents of a number of normal tissues and three experimental tumours in mice. Significant variations in the rate and degree of GSH depletion and recovery were observed among the normal tissues. Following a dose of 2.5 mmol kg$^{-1}$ BSO, GSH nadirs were reached by approximately 5 h for the liver and kidney, 8 h for the lung and bone marrow, 12 h for red blood cells (RBCs) and by 24 h for the heart. The degree of depletion was greatest for the kidney (80%), liver (74%) and bone marrow (83%), intermediate for the heart (54%) and lung (40%), and least for RBCs (13%). Recovery of GSH content was fastest for the liver followed in descending order by the kidney, the lung, the bone marrow, RBCs and the heart. In contrast, the rate and extent of GSH depletion and recovery showed considerably less variation among the 3 murine tumours. In the tumours GSH nadirs were reached by 10–12 h. The extent of depletion was about 55–65%. Recovery of GSH levels in the tumours required 48 h or more, a longer period than required by the liver, kidney and lung but shorter than that needed for the bone marrow, heart and RBCs. Attempts to preferentially deplete tumour GSH by exploiting the differences in recovery rates between normal tissues and tumours were only partially successful. Multiple BSO dosing at 16 h intervals allowed the liver to recover between doses, but the recovery in the kidney, lung and bone marrow was only partial and no recovery was seen in the heart. Finally, dose-depletion relationship investigations showed that, with the exception of the lungs, GSH depletion could be achieved in tumours with doses of BSO lower than those required for normal tissues.

It is now widely accepted that glutathione (GSH) plays an important role in the cellular defence against cytotoxic insults. Some tumour cells cultured *in vitro*, in particular those of human origin, were recently shown to contain extremely high levels of GSH (Biaglow *et al.*, 1983; Mitchell *et al.*, 1985). The possible relevance of GSH in cancer chemotherapy and the development of resistance during the course of treatment was emphasized by the findings that tumour cells made resistant to some anti-cancer drugs, e.g. melphalan, *cis*-platin and adriamycin, have increased cellular GSH concentrations (Suzukake *et al.*, 1982; Green *et al.*, 1984; Hamilton *et al.*, 1985). For these reasons much current interest has focused on techniques of reducing cellular levels of GSH prior to treatment with cytotoxic agents. The development of buthionine sulfoximine (BSO), a specific inhibitor of GSH synthesis, has removed much of the uncertainties due to unwanted side-effects that were associated with GSH depleting agents having less specificity. Recent *in vitro* studies using human tumour cell lines have shown that depletion of cellular GSH by BSO can indeed increase the cytotoxicity of a variety of anti-cancer drugs (Green *et al.*, 1984; Hamilton *et al.*, 1985; Crook *et al.*, 1986; Lee *et al.*, 1986).

Because of these promising developments, BSO in conjunction with chemotherapeutic drugs is likely to enter clinical trial in the near future. However, it is as yet not clear whether a therapeutic benefit can be obtained by selectively depleting tumour GSH contents while partly or wholly sparing the critical normal tissues. Consequently additional information on the *in vivo* use of BSO would greatly assist the planning of such clinical trials. Critical to such studies is a detailed knowledge of the GSH depletion kinetics of normal tissues compared to tumours. Furthermore, since the dose-limiting normal tissues may differ from drug to drug, information on a wide spectrum of critical normal tissues is clearly needed. In this study, we have investigated in detail the dose-response relationships and kinetics of GSH depletion by BSO in a range of normal tissues and tumours in mice.

Correspondence: F.Y.F. Lee.
Received 17 November 1986; and in revised form, 12 March 1987.

### Materials and methods

### *Mice and tumours*

All experiments were performed on 8–12 week-old inbred, female C3H/HeJ mice (Jackson Lab., Bar Harbor, ME). The murine sarcoma KHT (Kallman *et al.*, 1967) and RIF-1 (Twentyman *et al.*, 1980), and the mammary carcinoma 16C (Corbett *et al.*, 1978) were grown in the gastrocnemius muscle. Tumour-bearing mice were given BSO when tumours were between 250–350 mg.

### *Drug administration*

Buthionine sulfoximine (BSO), obtained in mixed enantiomeric form (DL-buthionine-SR-sulfoximine) from Sigma Chemical Co., was dissolved in phosphate buffer solution, pH 7.4, and injected i.p. at 0.01–0.04 ml g$^{-1}$ of mice.

### *Sample preparation*

Mice were sacrificed at various times following BSO treatment by cervical dislocation. Organs and tumours were then removed, rapidly washed in 10 mM 5-sulfosalicyclic acid/EDTA (5 mM) (Sigma), and dried on tissue paper. These were then frozen immediately by immersing in liquid nitrogen and stored at −70°C until analysis. To obtain bone marrow cells, femurs were removed, cleared from surrounding muscle, and washed with cold saline. Marrow was then expressed by flushing through with 1.0 ml cold saline using a needle and syringe. Marrows from the same group were pooled and syringed repeatedly to obtain a single cell suspension. The suspension then was diluted appropriately and nucleated cells were counted using a Coulter Counter/Channelyzer following lysis of RBCs with Zapoglobin II (Coulter). Aliquots of the original suspension were diluted with excess 3% glacial acetic acid to lyse unnucleated cells and were then centrifuged at 1000 *g* for 10 min. The resultant pellet was stored at −70°C until analysis.

Tissues were homogenized with 20 vol (w/v) of 20 mM 5-sulfosalicyclic acid (SSA). Bone marrow cells were homogenized with 200 μl SSA. Tissue or cell homogenates were centrifuged for 40 sec in an Eppendorf microcentrifuge. GSH in the SSA supernatant was derivatized using the fluorescent reagent monobromobimane (3-(bromoethyl)-2,5,6-trimethyl-1*H*, 7*H*-pyrazolo [1,2-*a*] pyrazole-1,7-dione; mBBR, Calbiochem., LaJolla, CA). Aliquots (180 μl) of the supernatant were pipetted into glass tubes containing 12 μl of N-ethylmorpholine (0.5 M in 20 mM KOH) and 2 μl of mBBR (50 mM in acetonitrile) and the sample immediately vortexed and stored in the dark at 4°C before analysis.

### *HPLC*

The isocratic HPLC technique used for the analysis of GSH was modified from the method of Minchinton (1984) and has been described previously (Lee *et al.*, 1986). Briefly, separation of GSH was carried out on Waters Radial-PAK reversed-phase bonded octadecylsilane cartridge columns (8 mm I.D., 5 μm diameter spherical particles). The mobile phase consisted of 23% acetonitrile in 40 mM ammonium phosphate, pH 7.2, containing 5 mM tetra-butyl-ammonium hydroxide, The effluent was monitored for fluorescence with 340 nm excitation and emission at >410 nm. The coefficients of variation were 7.1% and 6.4% at GSH concentrations of 0.5 mM and 5 mM respectively.

### Results

### *GSH concentrations in various normal tissues and three murine tumours*

The GSH contents of the normal tissues and tumours studied are listed on Table I. Among the normal tissues the liver was found to have the highest GSH concentration followed, in descending order, by the kidney, the lung, and the heart. Among the three murine tumours studied the KHT and RIF-1 sarcomas had similar GSH contents, whereas the 16C mammary carcinoma had significantly less GSH (Table I).

**Table I** The initial and nadir GSH concentrations in a range of normal tissues and 3 murine tumour models obtained from untreated mice and from mice treated with a single dose of 2.5 mmol kg$^{-1}$ BSO respectively. Data were calculated from 3 individual experimenets; 20–30 mice were used in each experiment

| *Tissue/ tumour* | *GSH levels in untreated mice* (*fmol cell*$^{-1}$ *or mmol kg*$^{-1}$)[a] | *GSH nadir concentrations after a single dose of BSO* (*fmol cell*$^{-1}$ *or mmol kg*$^{-1}$)[a] |
|---|---|---|
| Liver | 8.0±0.68[b] | 2.08±0.34[b] (26)[c] |
| Kidney | 2.83±0.33 | 0.567±0.06 (20) |
| Bone marrow | 0.3±0.05 | 0.05±0.01 (17) |
| Lung | 1.20±0.02 | 0.72±0.11 (60) |
| Heart | 1.11±0.07 | 0.511±0.09 (46) |
| RBCs | 0.142±0.021 | 0.124±0.015 (87) |
| KHT | 2.63±0.16 | 0.870±0.16 (33) |
| RIF-1 | 2.03±0.22 | 0.832±0.21 (41) |
| 16C | 1.26±0.09 | 0.529±0.09 (42) |

[a]The units were fmol cell$^{-1}$ for bone marrow and RBCs and mmol kg$^{-1}$ for all others; [b]±1 s.d. and [c]GSH concentration as percent of untreated controls is given in parentheses.

### *The time course of GSH depletion by single dose BSO*

Following a single dose of 2.5 mmol kg$^{-1}$ BSO, the GSH contents of the various normal tissues were depleted in a time-dependent manner (Figure 1). Kidney and liver showed the most rapid rates of depletion, with GSH levels reaching nadirs by ~5 h. Intermediate rates of depletion were seen in the lung and bone marrow with nadirs at 4–16 h and 8–12 h respectively. The heart was depleted the most slowly with a nadir in GSH at 24–72 h. The extent of GSH depletion following a single dose of BSO also differed significantly between tissues (Figure 1; Table I). The most severe depletion occurred in the liver, kidney and bone marrow; the heart and the lung showed intermediate depletion, and RBCs showed the least depletion. The recovery rates of GSH in the

**Figure 1** GSH contents as percent of initial concentrations in various normal tissues following a dose of 2.5 mmol kg$^{-1}$ BSO. Each datum point represents the average of 3 mice. Data are from 3 independent experiments. The initial GSH concentration for each normal tissue is given in Table I.

**Figure 2** GSH contents as percent of initial concentration in 3 murine tumour models following a dose of 2.5 mmol kg$^{-1}$ BSO. Each datum point is the average of 3 tumours; error bars indicate ±1 s.d. Data are from 2 independent experiments. The initial GSH concentration for each tumour is given in **Table I.**

various tissues following a single dose of BSO also differed considerably (Figure 1). Recovery, to pretreatment values, was most rapid for the liver (16 h) followed by the kidney (30 h), the lung (32 h), and bone marrow (72 h). Recovery was extremely slow for the heart (>96 h) and RBCs (>72 h). Furthermore, for the lung and in particular the liver and kidneys a pronounced 'overshoot' in GSH levels occurred during recovery, i.e. GSH concentrations rose significantly above those for untreated controls.

Unlike the considerable variation seen in normal tissues (Figure 1) the rate and extent of GSH depletion in the 3 murine tumours following a single dose of BSO (2.5 mmol kg$^{-1}$) were similar (Figure 2 and Table I). Recoveries, however, were more rapid in the KHT and RIF-1 tumours than in the 16 C mammary carcinoma (Figure 2).

*The dose-response relationship of GSH depletion by BSO*

The BSO dose-response relationship for all normal tissues and tumours was monitored at the time of the GSH nadir. Figure 3 shows the data for the normal tissues and Figure 4 those for the tumours. Great variations were observed between normal tissues in terms of sensitivity to BSO but little difference was seen in the three tumours. The maximum depletion that could be achieved with a single dose of BSO was again different for the various normal tissues, but similar for the tumours (Figures 3 and 4). BSO was least effective at depleting RBCs of GSH, intermediately effective for the heart and the lung and the most efficacious for the liver, kidney and bone marrow.

*GSH depletion by multiple doses of BSO*

To attempt to exploit further the differences in the recovery kinetics between some critical normal tissues and tumours, BSO was given every 16 h to mice bearing 16 C tumours. At this time GSH concentrations were at the nadir for tumours whereas in some normal tissues recoveries had already begun (Figures 1 and 2). Figure 5 shows the effects of giving 3 doses of BSO at 16 h intervals on the GSH contents of various normal tissues and 16 C tumours. With this treatment regimen, tumour GSH levels were depleted to 25% of untreated control values after 3 doses of BSO. The most rapidly recovering organ, the liver, recovered fully 16 h after the third dose. The lung, kidney and bone marrow recovered more slowly reaching 58, 46 and 38% of control by this time. The heart, however, showed no recovery, and was down to 20% of control GSH concentration. Finally, 16 C tumours also showed little recovery with GSH concentrations reduced to 30% of control at this time.

### Discussion

The present results demonstrate considerable diversity in the response of the various normal tissues and tumours to treatment with the specific GSH depleting agent BSO. Although investigations of the use of BSO as a tool in probing GSH metabolism and function, especially in cellular defense mechanism have been numerous, the details of GSH depletion and recovery kinetics following BSO treatment as well as the important question of dose selection *in vivo* have

**Figure 3** The dose-response relationship between BSO and GSH depletion in various normal tissues in mice. GSH contents are expressed as percent of initial concentration. Each datum point represents the average of 3 mice; error bars indicate ±1 s.d. Data are from 2 independent experiments.

**Figure 4** The dose-response relationship between BSO and GSH depletion in 3 murine tumour models. GSH contents are expressed as percent of initial concentration. Each datum point represents the average of 3 mice; error bars indicate ±1 s.d. Data are from 3 independent experiments.

**Figure 5** The effects of multiple BSO-dosing at 16 h intervals on the GSH contents of various normal tissues and the 16C mammary carcinoma. Arrows indicate the time at which BSO was administered. Data are from 2 independent experiments.

not yet been fully examined. A major aim of the present study was to attempt to delineate dosing and timing protocols which might result in differential depletion of tumour GSH content. In this respect, the data presented revealed clear differences between normal tissues and tumours which may be exploitable in the design of treatment regimens.

The ultimate goal in the manipulation of GSH levels is to improve the therapeutic index of a treatment regimen. However, a rational treatment strategy design will not be possible unless the relationship between the critical parameters of GSH depletion and the resultant biological effects are known. It is, for example, a matter of much current debate as to whether it is (i) the absolute GSH concentration following depletion, or (ii) the percent depletion of the initial GSH content which is the important parameter governing the chemo- and radiosensitization effects of GSH depletion. Since different tissues have vastly different steady state GSH levels, the effects of GSH depletion as indicated by these two parameters can differ dramatically. For example, the GSH content of the liver following depletion to 25% of its initial level is still greater than that of the untreated lung. In addition, a related question central to this debate is whether the various tissues have different GSH content for reasons of biological necessity or whether some tissues simply have large reserves of GSH that are not normally needed. The fact that tumour responses to some cytotoxic agents could be enhanced through GSH depletion whereas the cytotoxicity to the lung and bone marrow, two tissues with lower initial GSH content, were unaffected (see later), perhaps suggest a large degree of tissue dependence. If this were the case then the percent depletion for a particular tissue might be a more important factor. For this reason the percent depletion parameter primarily was used in the following discussion. Where necessary the absolute GSH levels can be deduced from the values of initial GSH content given in Table I.

In agreement with previous reports (Griffith and Meister, 1979; Moron et al., 1979; Hazelton and Lang, 1980), the GSH contents were found to differ widely among different normal tissues in the mouse. The highest concentration, found in the liver, was approximately 7 times that observed in the lung and the heart (Table I). Further the bone marrow has very low GSH content. Much less variation was seen in the 3 murine tumour models studied. The GSH contents in the tumours were relatively high in comparison with most normal tissues except for the liver and kidney (Table I; Griffith & Meister, 1979). Even higher GSH concentrations have been observed in human tumour xenografts grown in nude mice (Allalunis-Turner et al., unpublished results). Because of accumulating evidence implicating GSH in the cellular defence against anticancer agents (for review see Arrick & Nathan, 1984), much effort has been focused on reducing cellular GSH contents prior to treatment. BSO, a selective inhibitor of the GSH synthesis enzyme γ-glutamyl cysteine synthetase is considered superior to other depleting agents because of its specificity and lack of side-effects.

However, with a single high dose of BSO (2.5 mmol $kg^{-1}$), selective depletion of tumour GSH cannot be achieved (Figure 1 *vs*. Figure 2). Following such treatment, depletion was greater in the liver, kidney and bone marrow than in all 3 tumour systems studied (Table I). This is in agreement with the results of Minichinton and coworkers (1984) who observed patterns of GSH depletion in the liver and kidney as well as in tumours very similar to those reported here. In addition, attempts at preferential tumour depletion through multiple BSO exposures showed that selection through differential recovery rates was not entirely successful (Figure 5). In these multiple-dosing experiments GSH recovery in the liver was complete but recoveries in the lung, kidney and bone marrow were only partial. Furthermore, although with this treatment regimen, the GSH concentration of the 16C tumour could be severely reduced to 25% of pretreatment level, a similar reduction to 20% of initial concentration also was observed in the heart. This result was not unexpected because of extremely slow GSH recovery kinetics in this normal tissue (Figure 1). Since the heart is the dose-limiting tissue for the cytotoxic agent adriamycin (Lefrak *et al.*, 1973), caution should be exercised in any clinical trial considering the combination of adriamycin plus BSO so that cardiotoxicity is not enhanced through severe GSH depletion.

In contrast to the GSH kinetics investigations, the dose-response data (Figures 3 and 4) showed differences in GSH depletion between tumour and normal tissues which might be exploited to minimize adverse depletion in normal tissues, in particular the heart (Figures 3 and 4). The GSH content in the three tumour systems could be depleted considerably using lower doses of BSO than all normal tissues except the lungs. However, it remains to be seen whether such depletions are sufficient to enhance the tumoricidal effects of chemotherapeutic agents. If this were the case it may be possible to avoid or minimize GSH depletion in normal tissues by giving smaller doses of BSO. It should be noted that this approach will not be successful for the lung because of its greater sensitivity to BSO. However, by using multiple low dose schedules to take advantage of the faster recovery kinetics of the lung, coupled with the fact that depletion in the lung is less severe than in other tissues, adverse effects in the lung also may be avoided. Experiments to exploit this possibility are currently in progress.

The ability of BSO to potentiate the antitumour activity of chemotherapeutic agents has been demonstrated convincingly *in vitro* in human tumour lines for adriamycin (Hamilton *et al.*, 1985; Lee *et al.*, 1986), melphalan (Hamilton *et al.*, 1985; Green *et al.*, 1984), *cis*-platinum (Hamilton *et al.*, 1985; Lee *et al.*, unpublished) and activated cyclophosphamide (Lee *et al.*, unpublished results). *In vivo* chemosensitization also has been reported for cyclophosphamide (Ono & Shrieve, 1986; Tsutsui *et al.*, 1986), bleomycin (Tsutsui *et al.*, 1986), *cis*-platin (Tsutsui *et al.*, 1986), adriamycin (Lee & Siemann, in preparation) and melphalan (Alliet & Siemann, in preparation). However, ultimately the important question to be addressed is whether a therapeutic benefit can be achieved with combined cytotoxic agent-BSO treatment. While the data are limited, several studies have indicated that combining BSO with cytotoxic agents did not adversely affect normal tissue toxicity. For example, Russo and coworkers (1986) showed no effect of BSO on CFU-S survival and peripheral WBC counts following melphalan treatment. A similar result was found in our laboratory (Alliet and Siemann, in preparation). In Addition we have observed that a 2.5 mmol $kg^{-1}$ dose of BSO had no effect on the acute lethality of adriamycin (Lee & Siemann, in preparation) and no detectable effect on the lung toxicity of cyclophosphamide as measured by breathing rate or lung lavage protein assays (Allalunis-Turner *et al.*, unpublished results). Evidence so far is thus supportive of the idea that the therapeutic index of some chemotherapeutic drugs may be improved by BSO. It must be emphasized, however, that much more in-depth studies using normal tissue toxicity models best suited for each particular cytotoxic drug should be carried out before combination therapy with BSO can safely be used in patients.

Finally, if BSO is to be used in patients, the important question which needs to be addressed is how to monitor GSH depletion. The present results clearly indicate that no single normal tissue is representative of all others. The best strategy may be to monitor the GSH content of the critical dose-limiting tissue. As this is clearly not practical for all tissues, an alternative may be to monitor two or more tissues with different GSH response profiles, such as RBCs and bone marrow.

In conclusion, the present results indicate that the optimal use of BSO with chemotherapeutic drugs requires the detailed knowledge of the GSH kinetics in normal tissues and in tumours. For some normal tissues consideration probably will need to be given to both dose-timing and dose-selection in order to avoid severe GSH depletion.

This work was supported by NIH grants CA-36858, CA-11051, CA-38637 and CA-44127. MJAT was the recipient of a fellowship from the Alberta Heritage Foundation for Medical Research. The authors thank B. Granger for preparation of the manuscript.

### References

ARRICK, B.A. & NATHAN, C.F. (1984). Glutathione metabolism as a determinant of therapeutic efficacy: A review. *Cancer Res.*, **44**, 4224.

BIAGLOW, J.E., CLARK, E.P., EPP, E.R., MORSE-GUARDIO, M., VARNES, M.E. & MITCHELL, J.B. (1983). Non-protein thiols and the radiation response of A549 human lung carcinoma cells. *Int. J. Radiat. Biol.*, **44**, 489.

CORBETT, T.M., GRISWOLD, JR, D.P., ROBERTS, B.J., PECKHAM, J.C. & SCHABEL JR., F.M. (1978). Biology and therapeutic response of a mouse mammary adenocarcinoma (16/c) and its potential as a model for surgical adjuvant chemotherapy. *Cancer Treat. Rep.*, **62**, 1471.

CROOK, T.R., SOUHAMI, R.L., WHYMAN, G.D. & McLEAN, A.E.M. (1986). Glutathione depletion as a determinant of sensitivity of human leukemia cells to cyclophosphamide. *Cancer Res.*, **46**, 5035.

GREEN, J.A., VISTICA, D.T., YOUNG, R.C., HAMILTON, T.C., ROGAN, A.M. & OZOLS, R.F. (1984). Potentiation of melphalan cytotoxicity in human ovarian cancer cell lines by glutathione depletion. *Cancer Res.*, **44**, 5427.

GRIFFITH, O.W. & MEISTER, A. (1979). Glutathione: Interorgan translocation, turnover, and metabolism. *Proc. Natl Acad. Sci.*, **76**, 5606.

HAMILTON, T.C., WINKER, M.A., LOWE, K.G. & 7 others (1985). Augmentation of adriamycin, melphalan, and cisplatin cytotoxicity in drug-resistant and -sensitive human ovarian carcinoma cell lines by buthionine sulfoximine mediated glutathione depletion. *Biochem. Pharmacol.*, **34**, 2583.

HAZELTON, G.A. & LANG, C.A. (1980). Glutathione contents of tissues in the aging mouse. *Biochem. J.*, **188**, 25.

KALLMAN, R.F., SILINI, G. & VAN PUTTEN, L.M. (1967). Factors influencing the quantitative estimation of the *in vivo* survival of cells from solid tumors. *J. Natl Cancer Inst.*, **39**, 539.

LEE, F.Y.F., VESSEY, A.R. & SIEMANN, D.W. (1986). Glutathione as a determinant of cellular response to adriamycin. NCI monograph (in press).

LEFRAK, E.A., PITHA, J., ROSENHEIM, S. & GOTTLIEB, J.A. (1973). A clinicopathologic analysis of adriamycin cardiotoxicity. *Cancer*, **32**, 302.

MINCHINTON, A.I. (1984). Measurement of glutathione and other thiols in cells and tissues: A simplified procedure based on the HPLC separation of monobromobimane derivatives of thiols. *Int. J. Radiat. Oncol. Biol. Phys.*, **10**, 1503.

MINCHINTON, A.I., ROJAS, A., SMITH, K.A. & 4 others (1984). Glutathione depletion in tissues after administration of buthionine sulfoximine. *Int. J. Radiat. Oncol. Biol. Phys.*, **10,** 1261.

MITCHELL, J.B., MORSTYN, G., RUSSO, A. & CARNEY, D.N. (1985). *In vitro* radiobiology of human lung cancer. *Cancer Treat. Symposia,* **2,** 3.

MORON, M.A., DEPIERRE, J.W., MANNEVIK, B. (1979). Levels of glutathione, glutathione reductase and glutathione S-transferase activities in rat lung and liver. *Biochim Biophys. Acta,* **582,** 67.

ONO, K. & SHRIEVE, D.C. (1986). Enhancement of EMT6/SF tumor cell killing by mitomycin C and cyclophosphamide following *in vivo* administration of buthionine sulfoximine. *Int. J. Radiat. Oncol. Biol. Phys.*, **12,** 1175.

RUSSO, A., TOCHNER, Z., PHILLIPS, T. & 5 others (1986). *In vivo* modulation of glutathione by buthionine sulfoximine: Effect on marrow response to melphalan. *Int. J. Radiat. Oncol. Biol. Phys.*, **12,** 1187.